# Subcutaneous Levetiracetam Application Sustains Therapeutic Drug Levels

**DOI:** 10.1089/pmr.2020.0119

**Published:** 2021-05-21

**Authors:** Sophia Westphal, Caroline Hertler, David Blum, Markus Schettle

**Affiliations:** Competence Center Palliative Care, Department of Radiation Oncology, University Hospital Zurich, Zurich, Switzerland.

**Keywords:** levetiracetam, palliative care, seizure, subcutaneous application

## Abstract

We report on a patient suffering from seizures caused by cerebral metastases of adenocarcinoma of the lung. Initially, the patient was treated effectively with oral levetiracetam. As the disease progressed, oral intake was no longer possible. Since levetiracetam had controlled the patient's seizures well, the medication delivery mode was switched first to intravenous application, followed by a return to oral administration. After further deterioration, subcutaneous levetiracetam application was used to control epileptic symptoms while avoiding the sedating effects of subcutaneous midazolam. Subcutaneous levetiracetam allowed for stable seizure control in the end-of-life situation. Serum levels of levetiracetam were assessed for all application conditions and demonstrate that therapeutic drug levels can be reached by subcutaneous application. This report from a tertiary care center in Switzerland adds to the sparse but growing evidence base for the use of subcutaneous levetiracetam to manage seizures in palliative and end-of-life care.

## Introduction

Seizures occur in patients with primary or metastatic brain tumors with a prevalence of up to 56% of cases in the end-of-life situation,^1^ resulting in impaired quality of life due to the risk of trauma and the burden of medication dependency. Seizures, particularly status epilepticus, can eventually lead to aphasia or loss of consciousness, negatively impacting a patient's ability to interact with relatives and friends. Currently, newer generation antiepileptic drugs are used with increasing frequency to treat epileptic seizures in brain tumor patients because of the better interaction profile than first generation seizure medication. Among the new medication, levetiracetam has become a frequently used drug with a good tolerability profile and effective anticonvulsant properties for any type of seizure, including status epilepticus. The substance is available as oral or intravenous application. However, data on off-label subcutaneous application are derived mainly from case studies,^2–4^ and there is little data on therapeutic drug levels with this mode of application.

## Case Description

One of the latest publications on this subject, a systematic review and evidence-based guideline on the role of prophylactic anticonvulsants in the treatment of adults with metastatic brain tumors, does not recommend prophylactic antiepileptic drugs in seizure-free patients with or without surgical resection.^5^ In this study we describe a patient treated with levetiracetam for symptomatic brain metastases, and we report the drug levels for each application after the first 2 days of administration (Table 1). This is a reasonable interval to delay sampling so that a steady state metabolism can be achieved.^6^ The patient had no known kidney disease, and renal function was regularly measured, falling at all times in normal range.

**Table 1.** Application-Dependent Level of Levitiracetam

Date of application	21.9–02.12	03.12–07.12	08.12–12.12	13.12–22.12
Application	Enteral	Intravenous	Enteral	Subcutaneous
Dose	2 × 750 mg	2 × 750 mg	2 × 750 mg	1500 mg/24 hours
μmol/L (range 118–235)	141.5^a^	109.2^a^	n.a.	210.2

^a^ Trough level.

n.a., not available.

A 69-year-old woman suffering from metastatic pulmonary adenocarcinoma was admitted to our palliative care department with clinical deterioration caused by tumor progression. Since her initial diagnosis 2.5 years ago, she had experienced first-line surgery followed by four lines of chemotherapy and whole brain irradiation. Before admission, she had been diagnosed with leptomeningeal dissemination and several supra- and infratentorial metastases. The patient had not shown documented myoclonic seizure, but due to reduced alertness, a status postseizure could not be excluded. For future seizure prophylaxis, she was put on oral levetiracetam 750 mg BID, a treatment under which she was assessed with a therapeutic serum concentration of levetiracetam of 141.5 μmol/L (trough level; reference range 118–235 μmol/L).

After admission through the emergency department, the patient was temporarily substituted with intravenous application of medications, including levetiracetam, due to fluctuating consciousness. After 2 days, serum concentration for levetiracetam was measured and found to be minimally subtherapeutic with 109.2 μmol/L (trough level; reference range 118–235 μmol/L). The patient was asymptomatic for seizures under this treatment.

She stabilized clinically and was put back on oral medication based on prehospitalization doses. She was able to enjoy her family. Plans for a transition to a nursing home were discussed. However, due to the spreading leptomeningeal disease, the patient deteriorated neurologically and was subsequently unable to swallow oral medication. A levetiracetam rectal suppository application did not exist at the time and had the disadvantage of being an intimate intervention. As the patient was well controlled for seizures with oral levetiracetam, we decided a subcutaneous catheter would easily be manageable in the hospital as well as in the outpatient setting. In this case, it could increase comfort and reduce patient distress in the context of the progressive intracranial disease. Levetiracetam medication was given with 1500 mg for 24 hours (three ampullae of Keppra^®^ 500 mg/5 mL diluted in NaCl 0.9% B. Braun^®^ 100 mL) subcutaneously, based on previously published cases.^7^ No other medication was given in the syringe driver. After 2 days of continuous administration, we sampled the patient to ensure that serum concentrations of the drug were sufficient to treat the patient. With the established dose and the new method of application, serum concentrations of levetiracetam were in therapeutic ranges with 210.2 μmol/L (reference range 118–235 μmol/L). The patient never suffered a documented seizure during hospitalization. The subcutaneous catheter did not cause the patient discomfort, and the injection site showed no sign of erythema.

Continuous application of levetiracetam was, therefore, proven to be well tolerated and adequate to prevent seizures. Serum concentration analysis confirmed that subcutaneous application was sufficient to sustain therapeutic serum concentrations of the medication with the same dose as given orally or intravenously. Drug monitoring helped to control the efficacy of the treatment as clinical monitoring alone did not provide enough evidence. A switch to a sedating benzodiazepine-based seizure prevention was, therefore, not necessary. The patient remained seizure free for the remaining 9 days of life without need for sedation.

## Discussion

Seizures are an important symptom of cerebral manifestations in oncology, impairing quality of life at all phases of the course of disease, including during the end-of-life phase. In palliative care, oral medication is stopped when dominant fatigue or incapacity to swallow occurs. Usually, at this point, medication is switched to a subcutaneous application. For most antiepileptic medication, however, a liquid solution for intravenous application is not available. Thus, seizure control for palliative care patients is often achieved by subcutaneous midazolam, which carries a serious interaction profile. Benzodiazepine controls seizures but induces sedation, potentially contradicting a patient's wish to experience important moments in the last days of life.

Levetiracetam is a newer generation antiepileptic with few side effects and no significant interaction profile. It is used as monotherapy for focal or generalized seizures and licensed for intravenous application. Several case studies have assessed the tolerability of levetiracetam when given subcutaneously. In a patient with primary high-grade brain tumor, repeated applications of levetiracetam resulted in effective seizure control.^3^ Likewise, in a patient with squamous cell carcinoma metastasized to the brain, seizure control was achieved by continuous application of levetiracetam.^8^ In a larger retrospective case study analysis combined with a prospective observational audit, subcutaneous application was well tolerated and clinically efficient, especially when given as continuous application through a separate catheter to avoid local reactions. Only three of the reported cases controlled for serum levels.^2–4^

Intravenous and subcutaneous treatment with levetiracetam are more expensive than oral therapy. A patient's individual therapy goals should drive decisions about the application method. The decision about medication delivery mode must be made on a case-by-case basis.

Our case study confirms that subcutaneous application of the standard dose of levetiracetam resulted in clinical seizure control, sustaining therapeutic serum concentrations of the drug when given continuously for 24 hours. The application was well tolerated, with no local reaction and no sedative side effects (Table 1).

## Abbreviations Used

BID (bis in die): twice a day
n.a.: not available
